# Supplementary material for: MicroRNA-9 mediated the protective effect of ferulic acid on hypoxic-ischemic brain damage in neonatal rats
Source: PLoS One. 2020 May 29;15(5):e0228825. doi: 10.1371/journal.pone.0228825 (PMC7259979; doi:10.1371/journal.pone.0228825)
Supplement: S1 Checklist. The ARRIVE guidelines checklist — (DOCX) [file pone.0228825.s001.docx]

The ARRIVE Guidelines Checklist

Animal Research: Reporting In Vivo Experiments

Keli Yao ^a^, Qin Yang ^a^, Yajuan Li ^a^, Ting Lan ^a^, Hong Yu ^a, *^, Yang Yu ^a, *^

^a^ Department of Histology and Embryology, School of Basic Medical Sciences, Southwest Medical University, Sichuan Province, China

| ITEM RECOMMENDATION Section/paragraph | | | |
| --- | --- | --- | --- |
| Title | 1 | MicroRNA-9 Mediated the Protective Effect of Ferulic Acid on Hypoxic-Ischemic Brain Damage in Neonatal Rats | Title |
| Abstract | 2 | Neonatal hypoxic-ischemic brain damage (HIBD) is prone to cognitive and memory impairments, and there is no effective clinical treatment until now. Ferulic acid (FA) is found within members of the genus *Angelica*, reportedly shows protective effects on neuronal damage. However, the protective effects of FA on HIBD remains unclear. In this study, using the Morris water maze task, we herein found that the impairment of spatial memory formation in adult rats exposed to HIBD was significantly reversed by FA treatment and the administration of LNA-miR-9. The expression of miRNA-9 was detected by RT-PCR analyses, and the results shown that miRNA-9 was significantly increased in the hippocampus of neonatal rats following HIBD and in the PC12 cells following hypoxic-ischemic injury, while FA and LNA-miR-9 both inhibited the expression of miRNA-9, suggesting that the therapeutic effect of FA was mainly attributed to the inhibition of miRNA-9 expression. Indeed, the silencing of miR-9 by LNA-miR-9 or FA similarly attenuated neuronal damage and cerebral atrophy in the rat hippocampus after HIBD, which was consistent with the restored expression levels of brain-derived neurotrophic factor (BDNF). Therefore, our findings indicate that FA treatment may protect against neuronal death through the inhibition of miRNA-9 induction in the rat hippocampus following hypoxic-ischemic damage. | Abstract |
| INTRODUCTION | | | |
| Background | 3 | Neonatal hypoxic-ischemic brain damage (HIBD) is a severe disease that can cause irreversible neurological sequelae, such as cerebral palsy, mental deficiency, memory impairment and learning disabilities, and is often characterized by permanent neurological deficits. Thus, effective therapeutic agents which inhibit damage cascades activated after HIBD should be identified.  We established HIBD model used on P7 SD-rats, because P7 rats correspond to human neonatal, and the HIBD model was established by the classic method of Rice-Vannucci. | Paragraph 1 |
| Objective | 4 | we aimed to explore neuroprotective effect of FA on HIBD patients and further characterize the neuroprotective effect of FA on learning and memory ability, possibly via the downregulation of miR-9 following hypoxic-ischemic injury. | Paragraph 3 |
| METHODS | | | |
| Ethical statement | 5 | All animal experiments were performed under the protocol approved by the Animal Research Committee of Southwest medical university in accordance with the “Health Guide for the Care and Use of Laboratory Animals” which approved by Sichuan Experimental Animal Management Committee (Protocol Number: 202089). All surgery was performed under sodium pentobarbital anesthesia, and all efforts were made to minimize suffering. | Paragraph 5 |
| Study design | 6 | One hundred and sixty-eight seven-day-old Sprague-Dawley (SD) rats were provided by the Animal Department of Southwest Medical University (license number: SYXK (Sichuan) 2018-065, LuZhou City, China) and maintained under SPF conditions. The rats were divided into six groups randomly: Control group (28 rats), Sham group (28 rats), HIBD group (28 rats), HIBD+SF group (28 rats), HIBD+LNA group (28 rats).  P7: HIBD model establish, P21: drug treatment, P26, sacrifice rats and collection hippocampal tissues, P30: Morris water maze experiment. | Paragraph 7,8  Paragraph 13 |
| Experimental procedures | 7 | 1, HIBD model establishment: The left carotid artery of 7-day-old SD rats was permanently unilateral ligated and removed, and the pups were returned to their mothers for 1 h for recovery. Then, the pups were exposed in a hypoxic environment (8% O_2_ and 92% N_2_) for 2 h.  2, Intraperitoneally injected with 50 mg/kg SF: the drug concentration is calculated by the Animal equivalent dose calculation based on body surface area.  3, Anaesthesia via intraperitoneal injection of sodium pentobarbital.  4, 400 μM of LNA-miR-9 was stereotaxically microinjected into the lateral ventricle (1.0 mm posterior to bregma, -0.8 mm from the midline, and -3.5 mm deep from the dura) within 15 min. | Paragraph 4  Paragraph 7 |
| Experimental  animals | 8 | SD-rats. | Paragraph 4 |
| Housing and husbandry | 9 | 1, The rats maintained under SPF conditions.  2, The rats were housed 3–5 animals/cage and kept on a 10:14 h light-dark cycle, RT 22 ± 1˚C, and humidity of 55% ± 5%. | Paragraph 4 |
| Statistical methods | 10 | All data are expressed as the means ± standard error (SE). Differences between two groups were evaluated statistically by using unpaired Student’s t test. Differences among three or more groups were compared by analysis of variance (ANOVA) which with Tukey’s adjustment for multiple comparisons. P＜0.05 was considered statistically significant. | Paragraph 14 |
| RESULTS | | | |
| Numbers analysised | 11 | N=6 for RT-PCR and WB analysis in each group.  N=6 for HE-staining in each group.  N=10 for Morris water maze in each group. | Figure legend. |
| DISSCUSSION | | | |
| Interpretation/ scientific implications | 12 | In this study, we demonstrated for the first time that FA can improve learning and memory impairment caused by HIBD in rats. The neuroprotective effects of FA are likely the result of the regulation of miR-9 expression through increased release of BDNF. Further research is needed to clarify the mechanism underlying the effect of FA in the treatment of HIBD and make better use of it in the clinic; traditional Chinese medicine containing FA provides a theoretical basis for the treatment of HIBD. | Paragraph 1-4 |
| Funding | 13 | This work was supported by the Science and Technology Bureau of LuZhou City (2017LZXNYD-J30). | Acknowledgments |
