# Supplementary figures and images for: MicroRNA-9 mediated the protective effect of ferulic acid on hypoxic-ischemic brain damage in neonatal rats
Source: PLoS One. 2020 May 29;15(5):e0228825. doi: 10.1371/journal.pone.0228825 (PMC7259979; doi:10.1371/journal.pone.0228825)

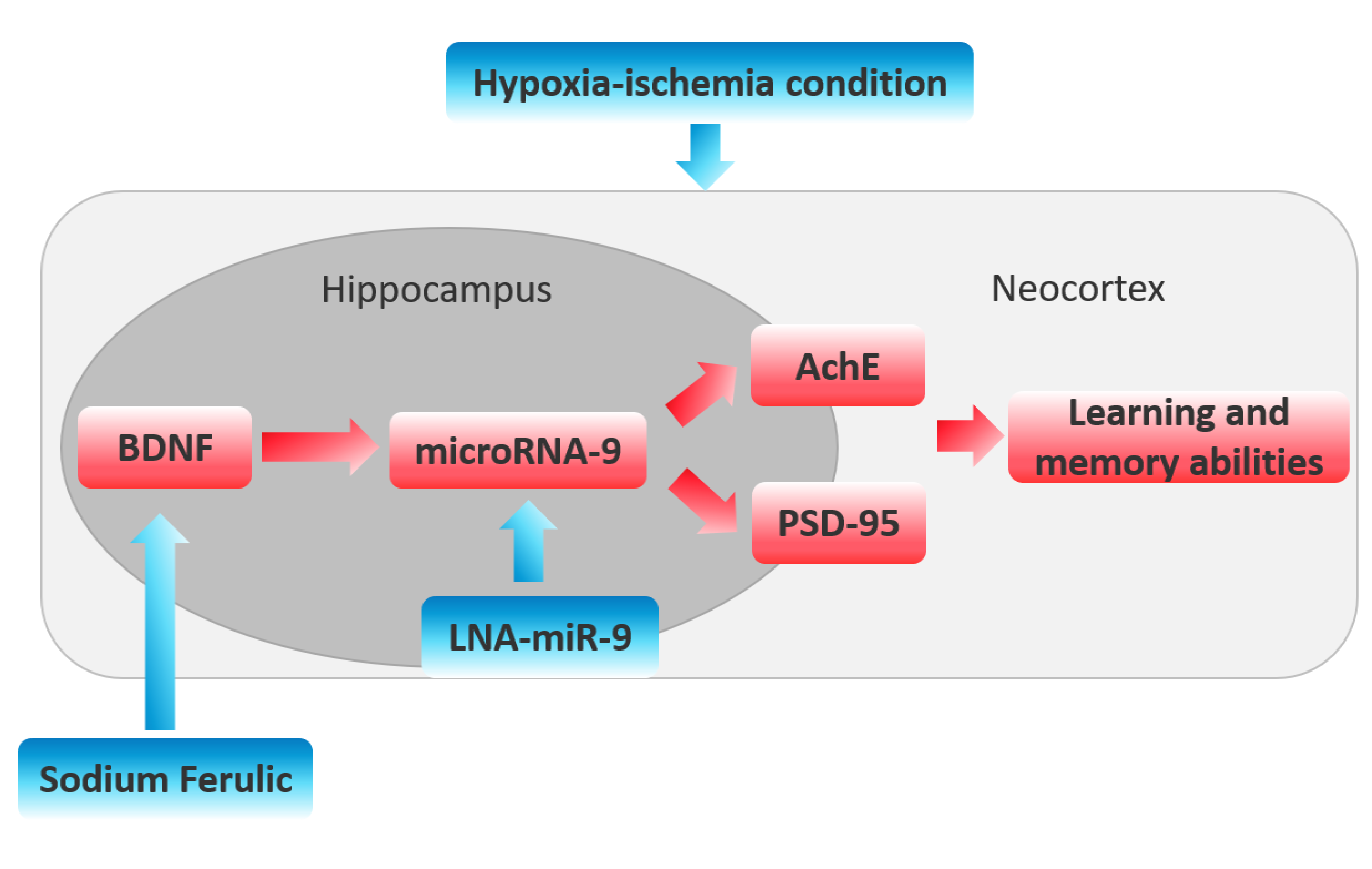

Supplement: S1 Fig — (TIF) [file pone.0228825.s002.tif]
