# Supplementary material for: MicroRNA-9 mediated the protective effect of ferulic acid on hypoxic-ischemic brain damage in neonatal rats
Source: PLoS One. 2020 May 29;15(5):e0228825. doi: 10.1371/journal.pone.0228825 (PMC7259979; doi:10.1371/journal.pone.0228825)

AchE

β-actin

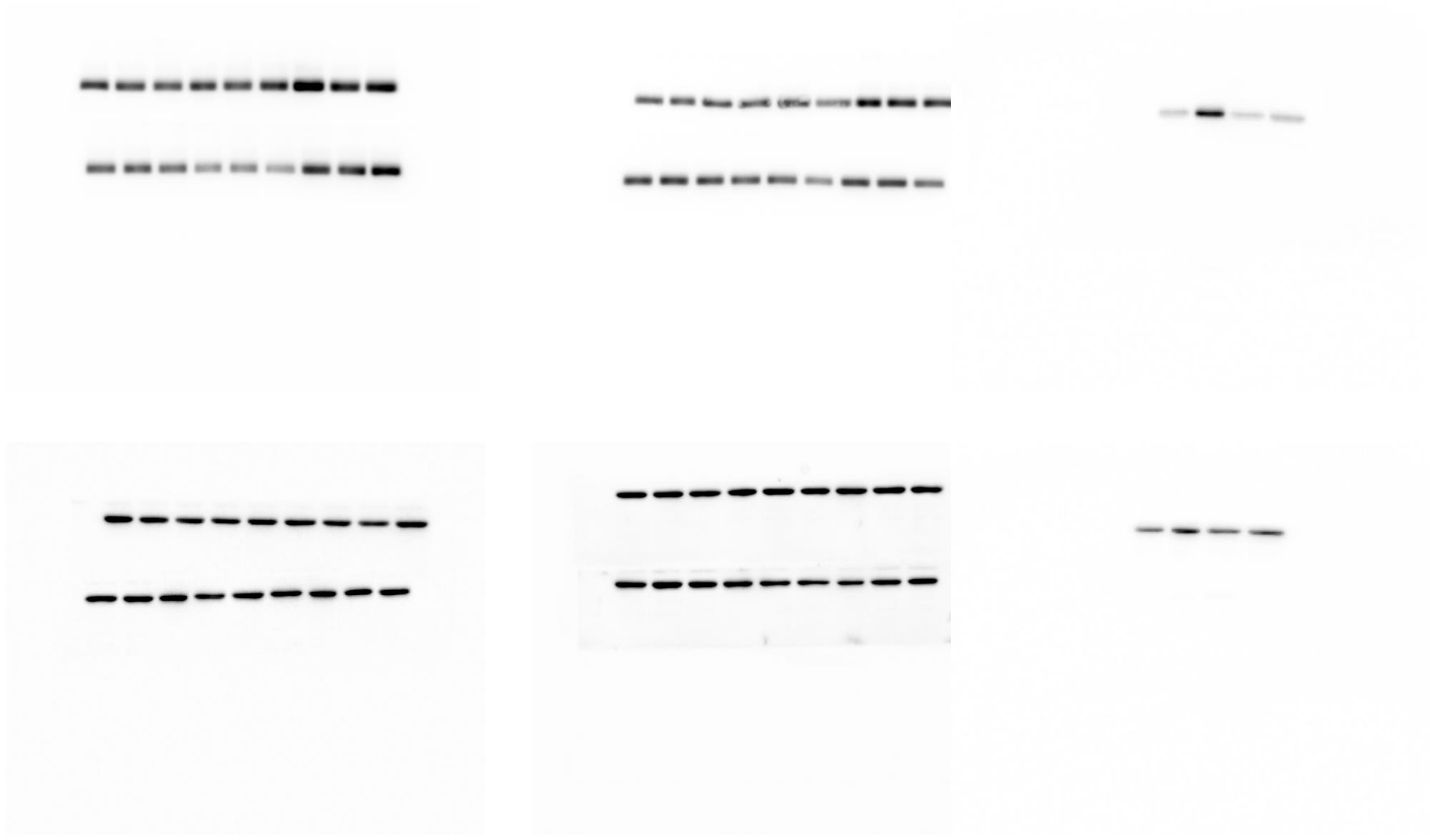

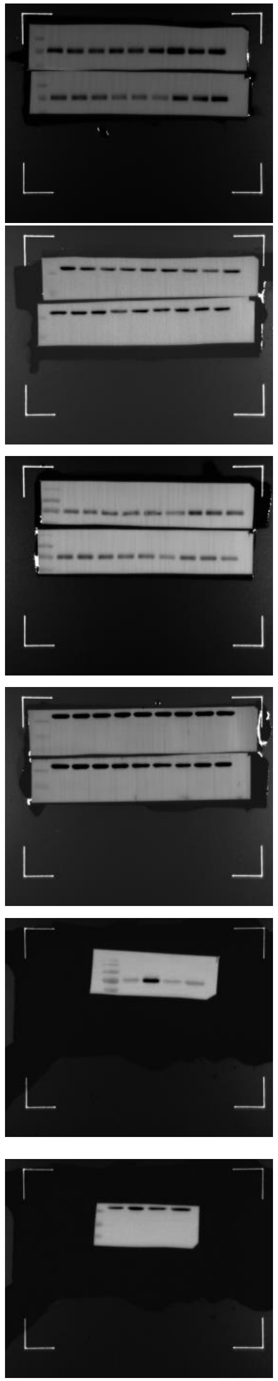

PSD-95

β-actin

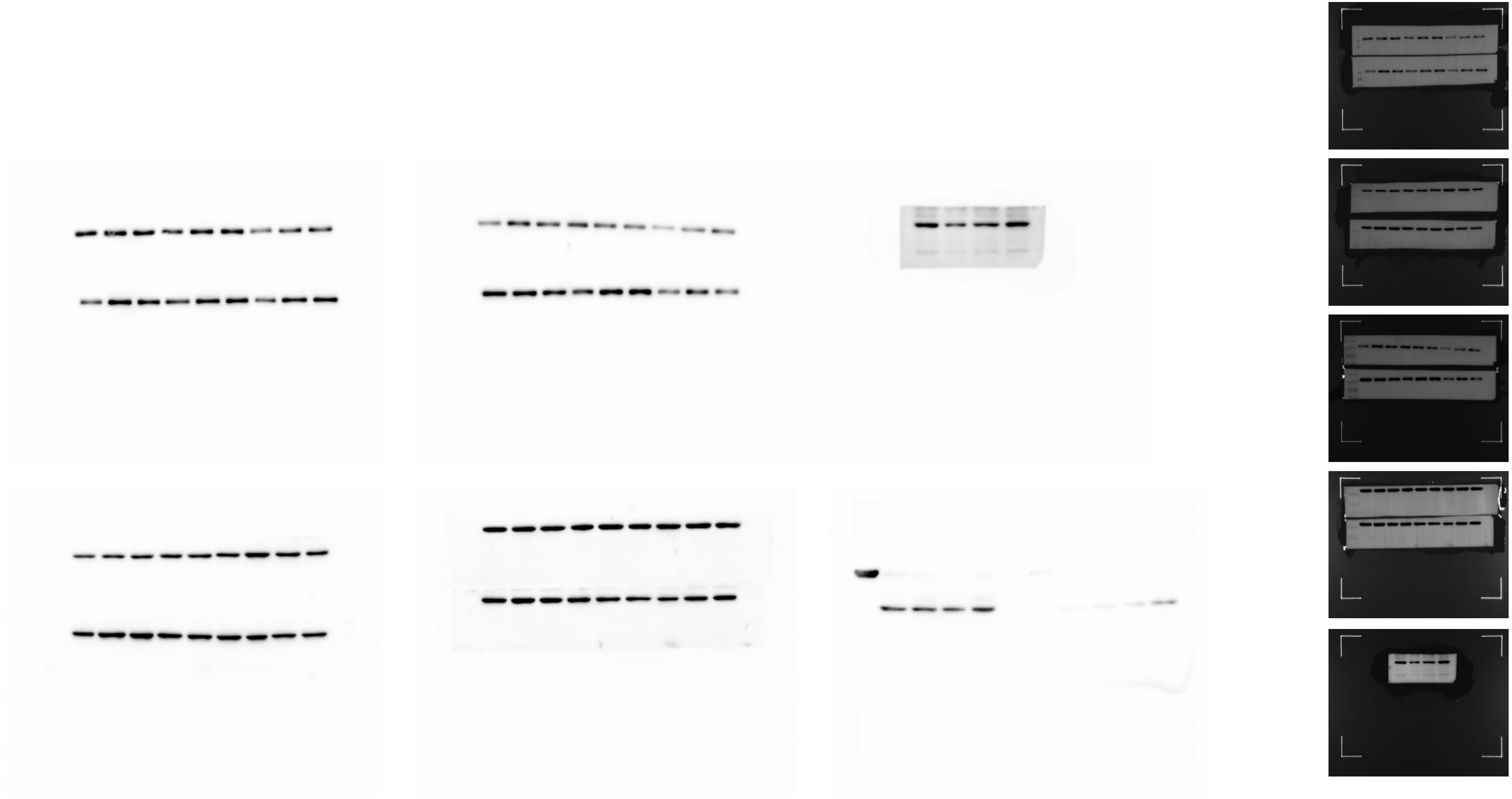

BDNF

β-actin

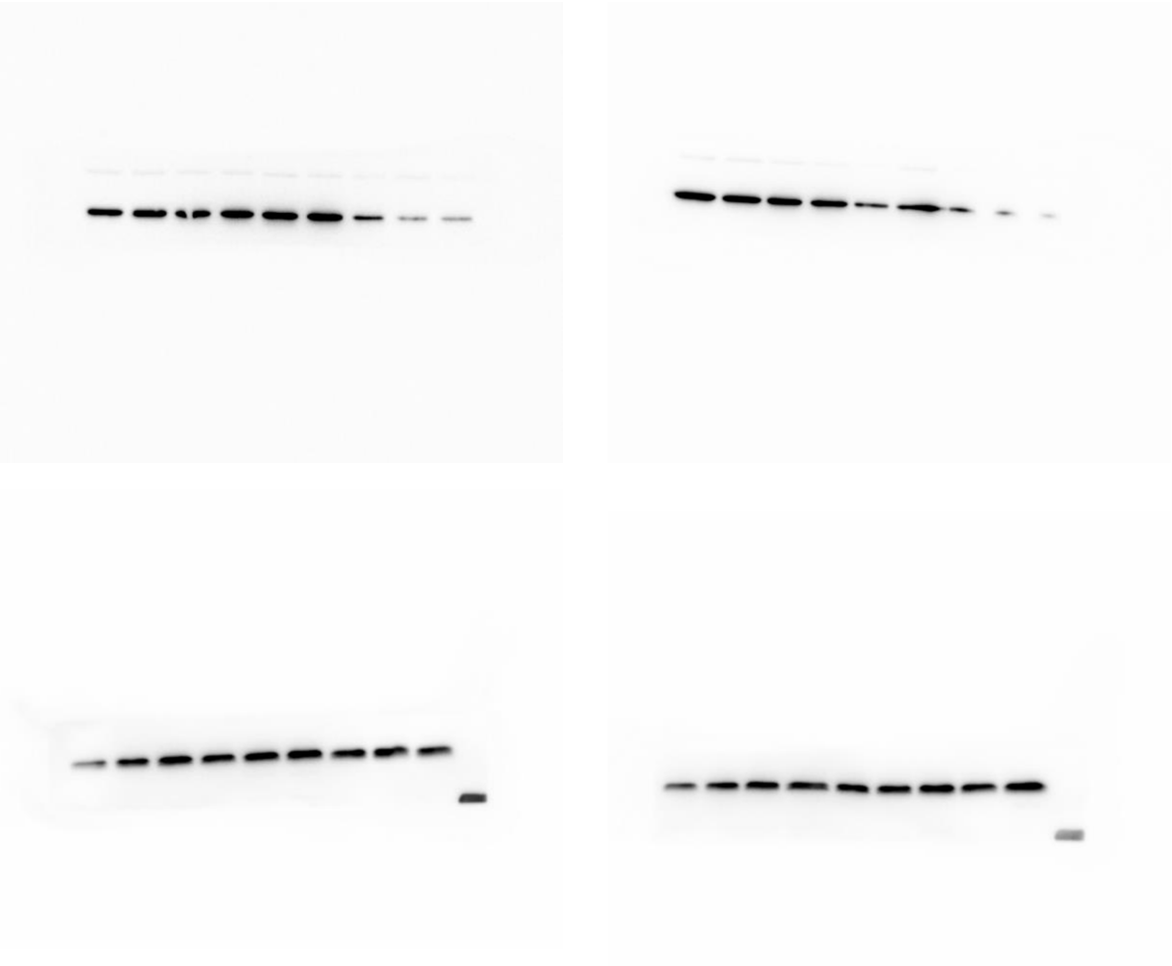

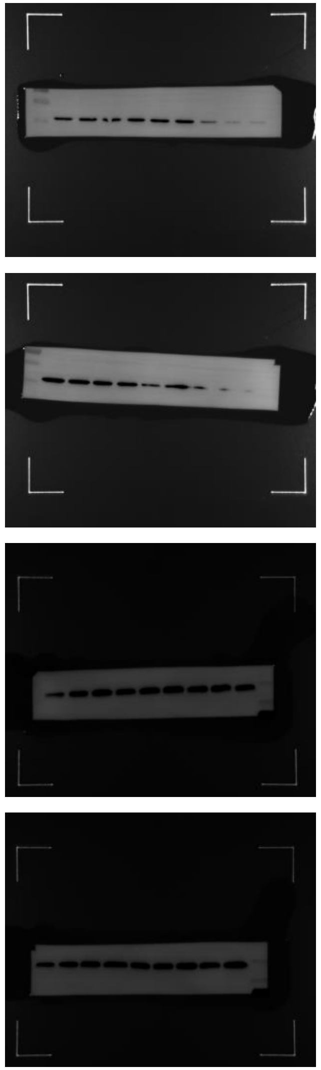

Supplement: S1 Original images — (PDF) [file pone.0228825.s003.pdf]
