# Supplementary material for: MicroRNA-9 mediated the protective effect of ferulic acid on hypoxic-ischemic brain damage in neonatal rats
Source: PLoS One. 2020 May 29;15(5):e0228825. doi: 10.1371/journal.pone.0228825 (PMC7259979; doi:10.1371/journal.pone.0228825)
Supplement: Attachment — Submitted filename: Response to Reviewers.docx [file pone.0228825.s004.docx]

PONE-D-20-01760

MicroRNA-9 Mediated the Protective Effect of Ferulic Acid on Hypoxic-Ischemic Brain Injury in Neonatal Rats

PLOS ONE

Dear Editor and Reviewers,

Thank you for taking the time to read my manuscript and thank you for your comments on my submission. Those comments are all valuable and very helpful for revising and improving our paper, as well as the important guiding significance to our researchers. We have studied comments carefully and have made correction which we hope meet with approval. Revise portion are marked in red in the paper. The main corrections in the paper and the responds to the reviewer’s comments are as following:

Reviewer #1:

1. Response to comment: Why we choose the dose of Sodium ferulic (50 mg/kg) for the intraperitoneally administration into the rats.

Response: The dose of Sodium ferulic (50 mg/kg) is calculated by the Animal equivalent dose calculation based on body surface area. The calculation procedure is:

AED (mg / kg) = Human does (mg / kg) × K_m_ ratio = 8.1 (mg / kg) × (37/6) = 50 mg / kg in rats. We have supplemented these data in the “methods and materials” section.

2. Response to comment: Why we choose to start FA at P21 rats.

Response: Since it is difficult to inject LNA-miR-9 with a stereotaxic instrument on the P7 damaged brain. 21-day-old rats are in the stage of high-speed brain growth and development, and drug intervention at this stage can observe the effect of FA and LNA-miR-9 on the neurological sequelae effectively. In addition, intraperitoneal injection of SF and brain stereotactic injection of LNA-miR-9 need to be synchronized at the same time, and the surgery of brain stereotactic injection is an invasive craniotomy, considering that rats are weaned at 21 days postnatally and have ability to eat independently, thus we choose to start drug treatment at P21 rats.

3. Response to comment: About “Authors should show the effect of FA administration in the cortex”.

Response: HE staining showed changes in the rat cerebral cortex and hippocampus, but the changes in the cerebral cortex were far less than those of in the rat hippocampus. Furthermore, since we mainly studied learning and memory impairment caused by HIBD, which is closely related to the hippocampus, the results of the cerebral cortex changes are only shown by HE staining. Relevant content about cerebral cortex have been supplemented in the paper.

4. Response to comment: About “indicate the total number of rats were used in the paper”.

Response: In this experiment, we used 168 rats, which have been supplemented in the “methods and materials” section.

5. Response to comment: About “indicate the total number of slices for each mouse used to perform the H-E staining, also reporting the bregma coordinate”.

Response: In this experiment, the brains were cut into 6-μm sections from Bregma -2.16 mm to -4.20 mm. Series of each 10 consecutive sections (intervals of 60μm) were cut and stained with HE.

Other changes: In the “Introduction” section and the “methods and materials” section, several statements that we made were more ambiguous than intended, and we have adjusted to the text to be clearer. We tried our best to improve the manuscript and made some changes in the manuscript. These changes will not influence the content and framework of the paper. And here we did not list the changes but marked in red in revised paper.

Special thanks to you for your good comments. We look forward to hearing from you regarding our submission and we would be glad to respond to any further questions and comments the you may have.

Kind regards,

Keli Yao

E-mail: 1129324624@qq.com
